# A new spider species of *Belisana* Thorell, 1898 (Araneae, Pholcidae) from Guizhou Province, south-western China

**DOI:** 10.3897/BDJ.12.e125111

**Published:** 2024-06-04

**Authors:** Bing Wang, Zhiyuan Yao, Xiaoqing Zhang

**Affiliations:** 1 College of Life Science, Shenyang Normal University, Shenyang, China College of Life Science, Shenyang Normal University Shenyang China

**Keywords:** Asia, daddy-long-legs, invertebrate, morphology, taxonomy

## Abstract

**Background:**

China exhibits remarkable diversity of the spider genus *Belisana* Thorell, 1898, with 62 species recorded to date. However, the largest number of *Belisana* species was found in Yunnan Province (23 ssp.), while only seven species were found in Guizhou Province.

**New information:**

In this paper, *Belisanawangchengi* sp. nov. as a new species is described from Guizhou Province, China.

## Introduction

The family Pholcidae C.L. Koch, 1850 is one of the most species-rich spider families, with 1969 extant species in 97 genera (World Spider Catalog 2024). *Belisana* Thorell, 1898, the second most abundant genus in Pholcidae, includes 148 species, 62 of which have been recorded from China. In recent years, a series of surveys of pholcid spiders have been carried out in China and a large number of new species have been reported (e.g. Yao et al. (2021), Lu et al. (2022), Zhao et al. (2023b), Yang et al. (2024a), Yang et al. (2024b), Zhang et al. (2024)). Nevertheless, these efforts focused on *Pholcus* Walckenaer, 1805 from northern and central China, with relatively few reports on *Belisana* from southern China. Zhu and Li (2021) described two new *Belisana* species from Xishuangbanna in south-western China. Zhao et al. (2023a) reviewed the *Belisana* spiders from Xishuangbanna and reported three additional new species. Yang et al. (2023) investigated the pholcid spiders in Guiyang, south-western China and reported four species, including one new member of *Belisana*. The aim of this article is to record a new species of *Belisana* from Tongren, a city at the foot of Fanjing Mountain in Guizhou Province.

## Materials and methods

Specimens were examined and measured with a Leica M205 C stereomicroscope. The left male palp was photographed. The epigyne was photographed before dissection. The vulva was dissected from the spider's body before being photographed and its soft tissue was dissolved in a 10% potassium hydroxide (KOH) solution. Images of the habitus, male palp, epigyne and vulva were captured with a Canon EOS 750D wide zoom digital camera (24.2 megapixels) mounted on the stereomicroscope mentioned above and assembled using Helicon Focus 6.7.1 image stacking software (Khmelik et al. 2005). Drawings were done with Procreate 5.0.2 (Savage Interactive Pty. Ltd.). All measurements are given in millimetres (mm). Legs are measured shown as: total length (femur, patella, tibia, metatarsus, tarsus). Leg segments were measured on their dorsal side. The specimens studied are preserved in 75% ethanol and deposited in the College of Life Science, Shenyang Normal University (SYNU) in Liaoning, China.

Terminology and taxonomic descriptions follow Huber (2005) and Yao et al. (2015). The following abbreviations are used in the descriptions: **ALE** = anterior lateral eye, **AME** = anterior median eye, **PME** = posterior median eye, **L/d** = length/diameter ratio; used in the illustrations: **aa** = anterior arch, **b** = bulb, **ba** = bulbal apophysis, **da** = distal apophysis, **e** = embolus, **ep** = epigynal pocket, **f** = flap, **pa** = proximo-lateral apophysis, **pp** = pore plate, **pr** = procursus.

## Taxon treatments

### Belisana wangchengi

Wang, Yao & Zhang sp. nov.

F5A15110-B9B1-5EC1-A080-1DF4A4AB211C

31B3B609-52B8-4E34-B2CC-C17C4AC563BF

#### Materials

- **Type status:** Holotype. **Occurrence:** recordedBy: Cheng Wang, Jiahui Gan, Chaojun Long, Hong Yao, Xufei Zhu; individualCount: 1; sex: male; lifeStage: adult; occurrenceID: 4D8A0EA9-EA92-518B-8596-1EE38113E9AC; **Taxon:** order: Araneae; family: Pholcidae; genus: Belisana; **Location:** country: China; stateProvince: Guizhou; municipality: Tongren; locality: Jiangkou County; verbatimLocality: Nujiang Town, Hekou Village, Shenjia Cave; verbatimElevation: 360 m a.s.l.; verbatimLatitude: 27°49.833’N; verbatimLongitude: 108°51.950’E; **Event:** samplingProtocol: Collected by hand; year: 2022; month: 11; day: 19; **Record Level:** institutionCode: SYNU-Ar00407
- **Type status:** Paratype. **Occurrence:** recordedBy: Cheng Wang, Jiahui Gan, Chaojun Long, Hong Yao, Xufei Zhu; individualCount: 3; sex: female; lifeStage: adult; occurrenceID: E4CD7AE1-5DB3-5368-9BBB-89EAFE6AA9E7; **Taxon:** order: Araneae; family: Pholcidae; genus: Belisana; **Location:** country: China; stateProvince: Guizhou; municipality: Tongren; locality: Jiangkou County; verbatimLocality: Nujiang Town, Hekou Village, Shenjia Cave; verbatimElevation: 360 m a.s.l.; verbatimLatitude: 27°49.833’N; verbatimLongitude: 108°51.950’E; **Event:** samplingProtocol: Collected by hand; year: 2022; month: 11; day: 19; **Record Level:** institutionCode: SYNU-Ar00408–00410

#### Description

**Figure 1.**
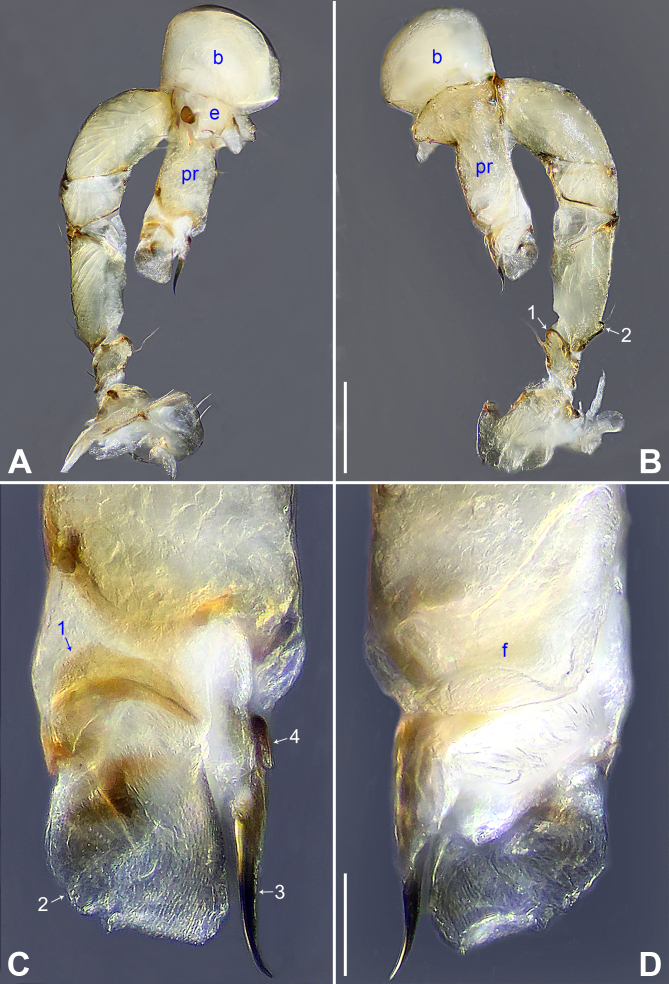
*Belisanawangchengi* sp. nov., male, holotype. **A, B** palp (**A** prolateral view **B** retrolateral view, arrow 1 points at ventral apophysis, arrow 2 points at retrolatero-proximal protrusion); **C, D** distal part of procursus (**C** prolateral view, arrow 1 points at prolatero-subdistal sclerite, arrow 2 points at distal membranous lamella, arrow 3 points at distal spine, arrow 4 points at sclerotised dorsal apophysis **D** retrolateral view). Abbreviations: b = bulb, e = embolus, f = flap, pr = procursus. Scale bars: 0.20 mm (**A, B**); 0.05 mm (**C, D**).

**Figure 2.**
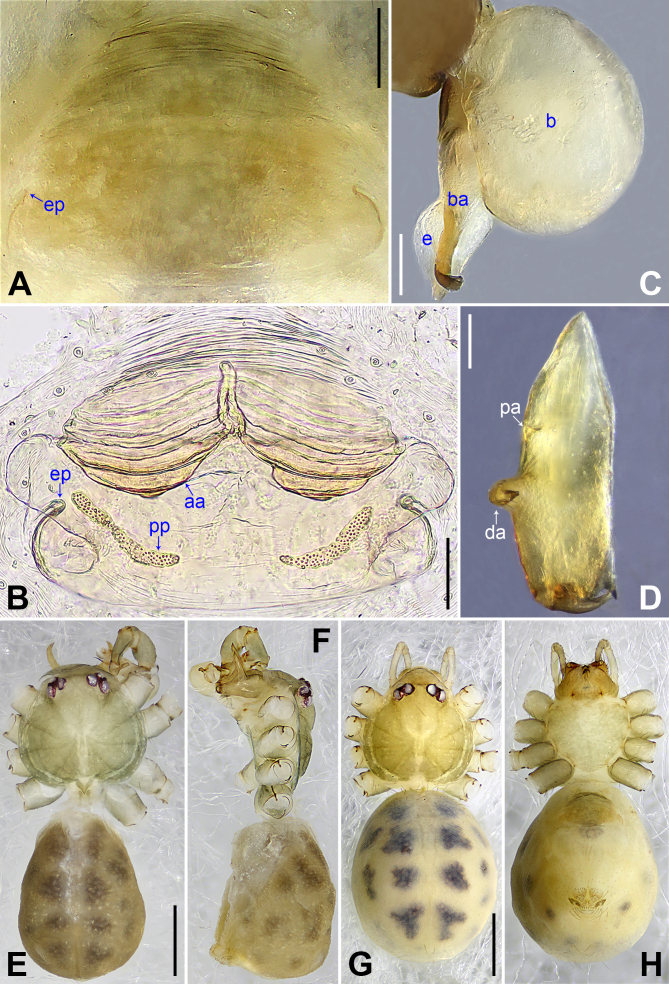
*Belisanawangchengi* sp. nov., male, holotype (**C–F**) and female, paratype (**A, B, G, H**). **A** epigyne, ventral view; **B** vulva, dorsal view; **C** bulb, prolateral view; **D** chelicerae, frontal view; **E–H** habitus (**E, G** dorsal view, **F** lateral view, **H** ventral view). Abbreviations: aa = anterior arch, b = bulb, ba = bulbal apophysis, da = distal apophysis, e = embolus, ep = epigynal pocket, pa = proximo-lateral apophysis, pp = pore plate. Scale bars: 0.10 mm (**A–D**); 0.50 mm (**E–H**).

**Figure 3.**
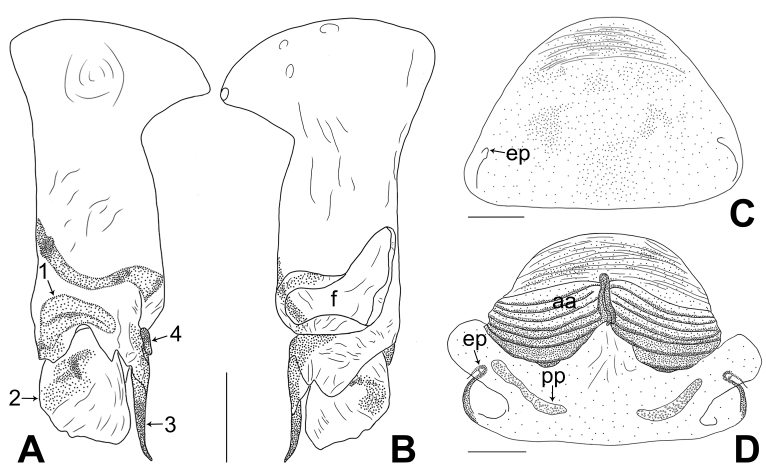
*Belisanawangchengi* sp. nov., holotype male (**A, B**) and paratype female (**C, D**) **A, B** procursus (**A** prolateral view, arrow 1 points at prolatero-subdistal sclerite, arrow 2 points at distal membranous lamella, arrow 3 points at distal spine, arrow 4 points at sclerotised dorsal apophysis **B** retrolateral view); **C** epigyne, ventral view; **D** vulva, dorsal view. Abbreviations: aa = anterior arch, f = flap, ep = epigynal pocket, pp = pore plate. Scale bars: 0.10 mm.

**Male** (holotype): Total length 2.06 (2.22 with clypeus), prosoma 0.76 long, 0.84 wide, opisthosoma 1.30 long, 0.88 wide. Leg I: 22.48 (6.11, 0.38, 5.58, 8.41, 2.00), leg II: 14.50 (4.06, 0.36, 3.68, 5.00, 1.40), leg III: 9.88 (2.75, 0.34, 2.36, 3.40, 1.03), leg IV: 12.73 (3.68, 0.35, 3.24, 4.40, 1.06). tibia I L/d: 63. Eye intervals and diameters: PME-PME 0.16, PME 0.08, PME-ALE 0.02, AME absent. Sternum as wide as long (0.58). Habitus as in Fig. 2E and F. Carapace of prosoma yellowish, with brown radiating marks; ocular area and clypeus brown; sternum brownish. Legs whitish, without darker rings. Opisthosoma yellowish, with dark brown spots. Thoracic furrow absent. Clypeus unmodified. Chelicerae (Fig. 2D) with pair of proximo-lateral apophyses and pair of curved distal apophyses (distance between tips: 0.36). Palp as in Fig. 1A and B; trochanter with ventral apophysis (as long as wide, arrow 1 in Fig. 1B); femur with small retrolatero-proximal protrusion (arrow 2 in Fig. 1B); procursus simple proximally, but complex distally, with prolatero-subdistal sclerite (arrow 1 in Fig. 1C and Fig. 3A), distal membranous lamella (arrow 2 in Fig. 1C and Fig. 3A) bearing proximal sclerotised part, curved distal spine (arrow 3 in Fig. 1C and Fig. 3A), sclerotised dorsal apophysis (arrow 4 in Fig. 1C and Fig. 3A) and nearly trapezoidal retrolateral membranous lamella (f in Fig. 1D and Fig. 3B); bulb (Fig. 2C) with hooked apophysis and simple embolus. Retrolateral trichobothrium on tibia I at 8% proximally; legs with short vertical setae on metatarsi; tarsus I with 22 distinct pseudosegments.

**Female** (paratype, SYNU-Ar00408): Similar to male, habitus as in Fig. 2G and H. Total length 2.16 (2.35 with clypeus), prosoma 0.72 long, 0.87 wide, opisthosoma 1.44 long, 1.25 wide. tibia I: 4.04; tibia I L/d: 54. Eye intervals and diameters: PME-PME 0.12, PME 0.09, PME-ALE 0.02, AME absent. Sternum as wide as long (0.60). Epigyne (Fig. 2A and Fig. 3C) simple and flat, brown, with pair of lateral pockets 0.41 apart. Vulva (Fig. 2B and Fig. 3D) with ridge-shaped anterior arch and pair of long (8 times longer than wide), curved pore plates.

#### Diagnosis

The new species resembles *B.yuhaoi* Yang & Yao, 2023 (Yang et al. 2023: figs. 2A–B, 3A–D and 4A–H) by having similar bulbal apophyses (Fig. 2C), but can be distinguished by procursus without prolatero-ventral lamella (Fig. 1C and Fig. 3A; vs. present), by procursus with nearly trapezoidal retrolateral membranous flap (Fig. 1D and Fig. 3B; vs. angular), by distal apophyses of male chelicerae pointing forwards (Fig. 2D; vs. downwards), by epigyne with pair of lateral pockets (Fig. 2A, B, Fig. 3C and D; vs. posterior) and by pore plates long and curved (8 times longer than wide, Fig. 2B and Fig. 3D; vs. nearly triangular).

#### Etymology

The specific name is a patronym in honour of the collector Cheng Wang (Tongren University); noun (name) in the genitive case.

#### Distribution

China (Guizhou, Tongren, Jiangkou County, Nujiang Town, Hekou Village, Shenjia Cave).

## Supplementary Material

XML Treatment for Belisana wangchengi
